# Neuroprotection in Neurodegenerative Disease: From Basic Science to Clinical Applications

**DOI:** 10.1155/2017/2949102

**Published:** 2017-02-28

**Authors:** Marta C. Monteiro, Michael D. Coleman, Eric J. Hill, Rui D. Prediger, Cristiane S. F. Maia

**Affiliations:** ^1^Faculty of Pharmacy, Center of Health Sciences, Universidade Federal do Pará (UFPA), Belém, PA, Brazil; ^2^Mechanisms of Drug Toxicity Group, School of Life and Health Sciences, Aston University, Aston Triangle, Birmingham B4 7ET, UK; ^3^Department of Pharmacology, Center of Biological Sciences, Universidade Federal de Santa Catarina (UFSC), Florianópolis, SC, Brazil

The limitations of current pharmacological treatments of neurodegenerative disease have led to the extensive investigation of novel compounds and nonpharmacological approaches to modify the course of these conditions, whilst reducing drug side effects. The definition of neuroprotection is complex and involves both preventing cell death and restoring function to damaged neurons, as well as restoring neuronal numbers. The development of drugs to slow or prevent the progression of neurodegenerative diseases might logically evolve from an improved understanding of the etiology and pathogenesis of these diseases. There have certainly been major advances in these areas over the past few years and the prospect for the introduction of “neuroprotective” therapies is much improved over previous years.

In this context, there is growing recognition that neuroinflammatory mechanisms might contribute to the cascade of events leading to neuronal degeneration observed in Alzheimer's disease (AD), Parkinson's disease (PD), stroke, and many other neurodegenerative diseases. Postmortem studies and experimental models have revealed an increase in activated glial cells and in concentrations of certain cytokines and interleukins in brain areas such as the hippocampus, cerebral cortex, substantia nigra, and striatum, which are all affected in neurodegenerative diseases. Therefore, the study of the role of neuroinflammatory mechanisms in the pathophysiology of neurodegenerative diseases and the potential of anti-inflammatory strategies for neuroprotection has also attracted the interest of many authors.

Lima and colleagues explored the patterns of microglial activation, astrocytosis, oligodendrocyte damage, myelin impairment, and Nogo-A immunoreactivity between 3 and 30 days after experimental striatal stroke in adult rats induced by microinjections of endothelin-1 (ET-1). They established the temporal evolution of these neuropathological events, which will be very relevant to future studies of neuroprotective drugs targeting neuroinflammation and white matter damage.

In the study by N. Lins et al., the authors investigated behavioral impairments and neuroinflammatory markers following the interaction between an intranasal arbovirus infection and the intrahippocampal injection of ME7 prion strain in C57BL/6. They observed that virus infection exacerbates the microglial inflammatory response to a greater degree in prion-infected mice, but this response was not correlated with hippocampal-dependent behavioral deficits.

Besides its regulatory effects in the light-dark cycle, melatonin is a hormone with neuroprotective, anti-inflammatory, and antioxidant properties. J. M. Mack and colleagues reviewed the literature about the potential role of the melatoninergic system in the pathogenesis and treatment of PD. The data available so far indicates that PD is associated with impaired brain expression of melatonin and its receptors MT1 and MT2. Exogenous melatonin treatment presented an outstanding neuroprotective effect in animal models of PD induced by different toxins. Melatonin might also potentially improve nonmotor symptoms commonly experienced by PD patients such as sleep and anxiety disorders, depression, and memory dysfunction.

Another important hormone is 17*β*-estradiol (E2), which, in addition to its multiple actions throughout the body and brain, exerts neuroprotective effects in both acute and chronic neurodegenerative diseases such as cerebral ischemia, traumatic brain injury, AD, and PD. R. Thakkar and colleagues investigated whether 17*β*-estradiol (E2), acting via the estrogen receptor coregulator PELP1, can exert anti-inflammatory effects in the ovariectomized rat and mouse hippocampus to regulate NLRP3 inflammasome activation after global cerebral ischemia (GCI). The authors showed that rats subjected to GCI had significantly higher NLRP3 inflammasome activation in the hippocampus compared to the sham animals that did not undergo ischemia. In addition, E2 treatment suppresses both expression and activation of the NLRP3 inflammasome at both days 1 and 3 after injury. PELP1 together with upstream inducers of NLRP3 inflammasome activation (P2X7 and TXNIP) was essential for the anti-inflammatory effect of E2 to regulate the NLRP3 inflammasome after GCI. These findings showed a new insight into the anti-inflammatory effect of E2 in the brain, suggesting that the NLRP3 inflammasome is a potential therapeutic target and E2 analogues or NLRP3 inflammasome selective inhibitors may have efficacy in the treatment of some neurodegenerative disorders, such as GCI.

Calcium is a second messenger and plays an important role in regulating a great variety of neuronal functions, such as release of neurotransmitters, synaptic plasticity, neuronal excitation, and gene transcription. Indeed, changes of intracellular free Ca^2+^ concentration ([Ca2+]i) may directly alter neuronal excitability. X. Liu and colleagues explored Ca^2+^ status as part of their investigation into the cardiovascular effects of H2S, which were due to decreased oxidative stress, via inhibition of NADPH oxidase activity in the rostral ventrolateral medulla (RVLM) of spontaneously hypertensive rats (SHR). The molecular mechanisms involved in this neuromodulator effect of H2S are not clear, although the effects, both exogenous and endogenous, of H2S in primarily cultured medullary neurons elevate [Ca2+]_i_ levels, mainly by increasing the calcium influx and mobilizing intracellular Ca^2+^ stores from ER from primarily cultured rat medullary neurons.

In HIV-associated neurocognitive disorders (HAND), the HIV-1 transactivator protein (Tat) activated from HIV-1-infected cells and cell membranes may lead to disruption of tight junctions (TJs) associated with astrocytes along the blood-brain barrier (BBB), which increase the permeability of this barrier and can lead to HAND. Y. Chen and colleagues investigated the relationship between occludin and amyloid-beta (A*β*) transfer receptors in human cerebral microvascular endothelial cells (hCMEC/D3) in the context of HIV-1-related pathology. The authors showed that HIV-1 Tat and the Rho inhibitor hydroxyfasudil (HF) had no significant effect on hCMEC/D3 cell viability. However, the HF significantly inhibited HIV-1 Tat-induced occludin dysfunction and regulated LRP1 and RAGE expression in hCMEC/D3 cells. Thereby, they suggested a potential protective role for HF in HIV-1 Tat-mediated BBB destruction and A*β* accumulation, revealing a new therapeutic strategy for reducing the A*β* burden in HAND.

Natural products have emerged as a source for potential agents, which might ameliorate CNS neurodegeneration. Whilst the antioxidant properties of the bee-derived plant resin propolis have been extensively reported, its effects on the behavioral/cognitive functions have been poorly described. C. C. S. D. M. Da Silveira et al. examined the neurobehavioral effects of yellow propolis, related to anxiety, depression, and spontaneous locomotor activity in rats. The authors also investigated mnemonic activity as well as antioxidant properties of the extract, since both behavioral and cognitive disorders may manifest themselves in the course of the neurodegenerative diseases as symptoms or comorbidities. The authors concluded that yellow propolis elicits antioxidant activity associated with anxiolytic, antidepressant, and cognitive enhancer properties. Therefore, yellow propolis shows some promise product from natural sources in the therapy of the central nervous system disorders, as neurodegenerative diseases.

It is well documented that oxidative stress plays a pivotal role in neurodegenerative diseases pathology, particularly with AD. Another feature related to AD is the occurrence of dementia, in which the cholinergic neuronal enzymatic activity [i.e., acetylcholinesterase (AChE) and butyrylcholinesterase (BuChE)] is involved in the optimal impulse transmission at cholinergic synapses. The AChE/BuChE upregulation promotes excessive degradation of the neurotransmitter acetylcholine in the synaptic terminals, which leads to cognitive dysfunctions. In fact, the treatment of AD symptoms related to cognitive decline is based on anticholinesterase activity modulation. D. Załuski and R. Kuźniewski investigated the biological activity of the 12 extracts of* Eleutherococcus* species related to both neurodegenerative mechanisms (i.e., oxidative stress and increased cholinesterase activity). Their results indicated that bioactive compounds from* Eleutherococcus *species are a promising source for the protection or treatment of neurodegenerative diseases, since the extracts were able to inhibit both AChE and BuChE avoiding feedback loop, allied to the antioxidant activity that in turn may ameliorate the cognitive dysfunction and reduce the oxidative damage, respectively.

Whilst it is clear from the studies included in this special issue that many advances have been made in basic research directed at improving the symptoms of neurodegenerative diseases, there are many key issues still to be overcome. Firstly, we remain ignorant of the genesis of the major neurodegenerative diseases in individuals, although the study of gene expression in human groups who are highly susceptible to AD such as those with Down's syndrome is a notable exception. Secondly, this special issue also illustrates that the mechanisms at play in neurodegenerative diseases are clearly multifactorial in terms of their impact on CNS functionality, with contributions from the immune system, antioxidant status, changes in gene expression, and epigenetic impacts on enzymatic function. Often, a single targeted therapeutic intervention will successfully repair a particular issue such as anti-inflammatory and antioxidant status, only for this apparent advance to have little or no impact on the genesis and progress of the neurodegenerative condition. Thirdly, there also remains the issue of suitability of appropriate models employed for the assessment of possible therapeutic initiatives. Whilst there are several animal models for various human neurodegenerative conditions, their record in providing relevant agents which are then successful in clinical trials has been problematic and in vitro models are very much in their infancy.

Overall, it is imperative that we continue to progress in our search for appropriate models, either in vivo or in vitro, which can adequately and faithfully assess integrated therapeutic initiatives, which can do much more than can be done at the moment, that is, simply treating the symptoms of these conditions and managing an inevitable decline. It is necessary to devise treatment regimens, which not only halt the progress of these conditions, but also one day prevent their appearance and even restore full function in those who have been afflicted by them. The progress reported in this special edition suggests that, in the future, achieving these aims might be a distant prospect but not an unattainable one.

*Marta C. Monteiro* *Michael D. Coleman* *Eric J. Hill* *Rui D. Prediger* *Cristiane S. F. Maia*

